# Desulfurization of Morupule Coal with Subcritical Aqueous Ethanol Extraction

**DOI:** 10.1002/open.202200046

**Published:** 2022-08-10

**Authors:** Fiona M. Nermark, Mmilili M. Mapolelo, James Darkwa, Ola F. Wendt, Charlotta Turner

**Affiliations:** ^1^ Centre for Analysis and Synthesis Department of Chemistry Lund University P O Box 124 22100 Lund Sweden; ^2^ Department of Natural Resources and Materials Botswana Institute for Technology Research and Innovation Private Bag 0084 Gaborone Botswana

**Keywords:** desulfurization, Morupule coal, pressurized liquid extraction, subcritical water extraction, sulfur

## Abstract

Coal combustion greatly contributes to global emissions of toxic gases into the atmosphere, with sulfur emissions as one of the prominent pollutants in addition to carbon dioxide. Nevertheless, Botswana utilizes Morupule's sub‐bituminous coal with average sulfur and ash contents, as determined in this study being 1.9 and 24.4 % by weight with an average calorific value of 22 MJ Kg^−1^ to generate electricity. We report an optimized extraction method for reducing total sulfur in Morupule coal from 1.9±0.2 to 0.43±0.02 wt.% at optimum conditions of ethanol/water (90/10, v/v %) at 129 °C (105 bars) in 10 minutes. A Box–Behnken experimental design was employed to select the optimal conditions of temperature (100–180 °C), water proportion in ethanol (10–90, v/v %) and extraction time (10–30 minutes), thus reducing the total sulfur under these mild conditions compared to conventional extraction. The optimized conditions were however not efficient in removing ash.

## Graphical abstract

**Subcritical aqueous ethanol** was utilized to reduce total sulfur in Morupule coal. The ratio of ethanol to water, extraction time and temperature were examined in a Box–Behnken design. The optimal conditions for desulfurization were extraction with 90 % ethanol for 10 minutes at 129 °C and 105 bars. A 79 % reduction in total sulfur was achieved.

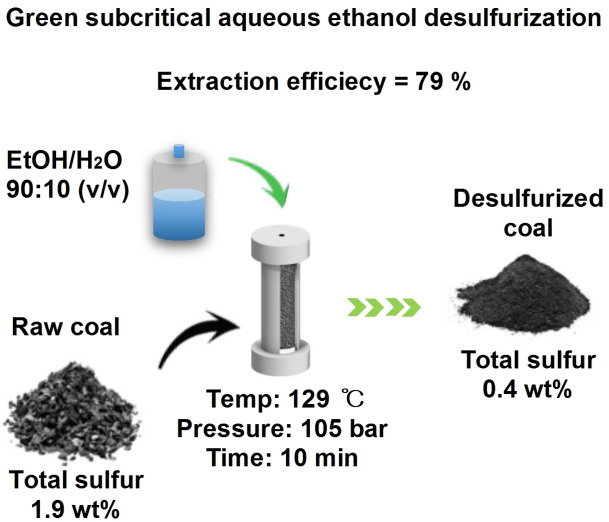

## Introduction

Coal is widely used for the generation of electricity and it is also used for the production of high‐value chemicals through coal gasification, liquefaction, and coke production.[^1^, ^2^] Combustion of coal is accompanied by the emission of toxic gases such as NO_x_ and SO_x_, as well as the release of toxic metals^[3]^ and organic pollutants^[4]^ The mineral matter present as ash after combustion as well as the sulfur content of coal, thus greatly limit its utilization. The mineral matter decreases the calorific value of the coal and the silicates and aluminosilicates present in the ash cause fouling, slagging and clinkering of heaters, furnaces and turbines during combustion, thereby resulting in the corrosion of equipment thereby reducing their lifespan.[^5^, ^6^] The sulfur content in coal can be used as an indicator of the acid dew point of flue gas,^[7]^ and in the steelmaking industry, sulfur is the most harmful element in the remaining steel since it reduces the mechanical strength and causes the steel to become brittle.^[8]^ Furthermore, in addition to CO_2_, the major atmospheric pollutant associated with coal combustion is sulfur dioxide, which causes the formation of acid rain.^[9]^ Thus, it is both economically and environmentally important to reduce the amount of ash and sulfur in coal before its utilization.

Coal demineralization and desulfurization occur primarily by physical methods such as gravity separation and flotation at the mine sites.^[10]^ However, gravity separation and flotation are not very effective in removing organic sulfur and finely dispersed inorganic sulfur and minerals that are commonly incorporated into the organic matter of coal.^[11]^ As a result, it is a challenge to effectively reduce inorganic sulfur in addition to organic sulfur using only gravity separation or flotation. The use of microbes (microbial desulfurization) such as *Acidithiobacillus ferrooxidans* has been studied, with reports showing an overall desulfurization of 79.7 % with organic sulfur reduced from 3.8 % w/w to 0.59 % w/w after 14 days (Rout et al., 2021).^[12]^ The disadvantages of microbial desulfurization are the pH sensitivity of the microbes and extended treatment time. Therefore, chemical methods have been explored. Ionic aqueous acid/base solutions such as hydrochloric acid, nitric acid, hydrofluoric acid, ammonia or sodium hydroxide have been used to leach out organic and inorganic sulfur from coal.^[13]^ Organic solvents such as light cycle oil,^[14]^ 1‐methylnaphthalene, N‐methyl‐2‐pyrrolidone (NMP), carbon disulphide, tetralin ethylenediamine, or quinoline have also been used,[^15^, ^16^, ^17^] and the remaining coal contains reduced inorganic matter, thereby minimizing emissions of trace metals and toxic particulate matter during combustion.^[18]^ Yue et al. (2022) investigated the desulfurization effect of UV–H_2_O_2_ under different sodium hydroxide (NaOH) and oxalic acid (OA) dosages on Lingshi high sulfur coal. They obtained 56 wt.% reductions with OA and 53 wt.% reductions with NaOH of the total sulfur in coal.[^19^, ^20^] Chemical desulfurization is certainly more efficient compared to physical desulfurization and relatively faster compared to microbial desulfurization. However, the main concern with chemical desulfurization is the reagents used because of their toxicity to the environment. In general, there is a trend towards utilization of greener extraction solvents in combination with techniques such as supercritical fluid extraction (SFE) and subcritical water extraction (SWE).[^21^, ^22^, ^23^] These approaches have for instance been explored in the pharmaceutical and food industries.[^24^, ^25^, ^26^]

In SWE, water is heated to temperatures above its boiling point and it is contained at high pressure, resulting in decreased relative permittivity, surface tension and viscosity whereas its diffusivity increases.[^27^, ^28^] Under subcritical conditions, water may act as an organic solvent with similar dielectric constant to methanol (ϵ=33 at 25 °C) at 220 °C or ethanol (ϵ=24 at 25 °C) at 270 °C.^[29]^ SWE has been widely used for the extraction of organic compounds such as phenolic compounds in fruits and vegetables.[^30^, ^31^] Nuapia et al. demonstrated the application of subcritical water for the recovery of macro‐nutrients and micro‐nutrient elements from dried leaf powder of *Moringa oleifera* ^[32]^ and Meng and Cheng used it for the simultaneous extraction of phenolic compounds and inorganic elements from *Erigeron breviscaous*.^[33]^ Supercritical fluids have also been used for coal desulfurization and early work reported the removal of both inorganic and organic sulfur compounds from coal at temperatures above 350 °C.[^21^, ^22^, ^23^]

A systematic method for desulfurization through extraction with a mixture of ethanol and water in varying proportions at temperatures above 350 °C has been reported by Azzam and Lee,^[21]^ but the optimized conditions resulted in the solvent being reactive that in turn degraded the coal as seen from C−C bond cleavage in the coal structure. Therefore, milder conditions are needed. Here we present an optimized extraction method that reduces the total sulfur from 1.9 % to 0.4 % by weight, with a 79 % desulfurization efficiency in raw coal. The extraction method uses subcritical aqueous ethanol at a temperature that is not expected to thermally degrade the coal prior to gasification or combustion. For maximum reduction of mineral matter in coal and desulfurization with aqueous ethanol, the effect of parameters such as extraction temperature, extraction time, and amount of organic co‐solvent have to be investigated. One way to do so is to use a response surface methodology (RSM). RSM is useful during method development and optimization of processes to establish the relationship between a set of quantitative experimental variables and one or more response variables. With the help of the optimizer function in the tool, optimum parameter are predicted.^[34]^

## Results and Discussion

The coal was first characterized by proximate and ultimate analysis, calorific value and X‐ray fluorescence (XRF) spectroscopy. The coal is ranked as low sulphur sub‐ bituminous coal according to the American Society for Testing and Material (ASTM) standard classification with a calorific value of 22.3 MJ kg^−1^ (Table 1). However, the coal has high ash content of 24.4 wt.%. As already mentioned, a high ash content in coal is both environmentally^[35]^ and economically undesirable due to the solid waste produced and the fouling, slagging and corrosion of reactors.^[36]^ The main constituents of the Morupule coal ash in our study turned out to be SiO_2_ at 42.5 wt.%, and Al_2_O_3_ 27.7 wt.%. The other oxides with significant abundance were Fe_2_O_3_ (7.6 wt.%), CaO (10.1 wt.%), SO_3_ (5.7 wt.%), and TiO2 (3.3 wt.%). There were also trace amounts of MgO (0.9 wt.%), K_2_O (0.5 wt.%), Na_2_O (0.4 wt.%), MnO_2_ (0.1 wt.%), and P_2_O_5_ (1.1 wt.%). The scope of this work was limited to exploring overall reduction of ash content rather than individual mineral oxides.

**Table 1:** Proximate and ultimate analysis of the raw coal under study, determined following ASTM methods for coal and coke. n=3.

Parameter	Weight percent [%]
Moisture^[a]^	3.6±0.1
Volatile matter^[b]^	23.8±0.3
Fixed Carbon^[c]^	48.2±0.2
Ash^[d]^	24.4±0.04
Carbon^[e]^	56.4±0.8
Hydrogen^[e]^	4.6±0.1
Oxygen (by difference)	8.0±0.6
Total sulfur^[f]^	1.9±0.2
Other inorganics (by difference)	27.9±0.3
Calorific value^[g]^ (MJ/Kg)	22.3±0.2

[a] ASTM D3173,^[38]^ [b] ASTM D3175,^[39]^ [c] ASTM D5142,^[40]^ [d] ASTM D3174,^[41]^ [e] ASTMD5373,^[42]^ [f] ASTMD4239,^[43]^ [g] ASTMD5865.^[44]^

The aim of this study was to develop and optimize a greener extraction method to reduce the sulfur and mineral matter (ash) content of Morupule raw coal. The coal would be treated prior to its gasification to produce synthesis gas (syngas=CO+H_2_). In other words, what is needed is an overall reduction in mineral matter and sulfur under conditions that do not thermally degrade the coal. A method based on a subcritical solvent mixtures of water and ethanol was optimized at temperatures between 60–180 °C by the design of experiments (DoE) using a Box–Behnken design.^[37]^

### Model validity of the Box–Behnken design

**Figure 1:**
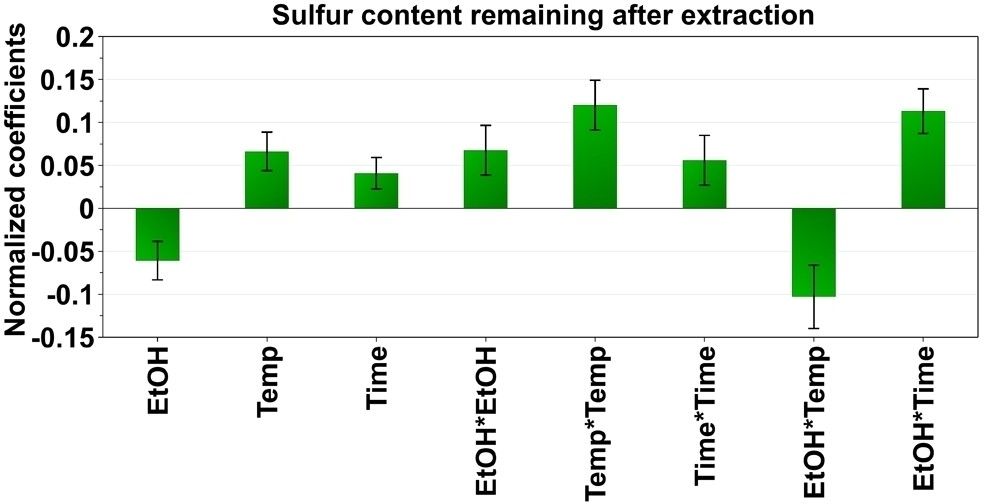
Coefficient plots from the Box–Behnken design showing the influence of different extraction parameters on the total sulfur content (wt.%) remaining in the extracted coal.

**Figure 2:**
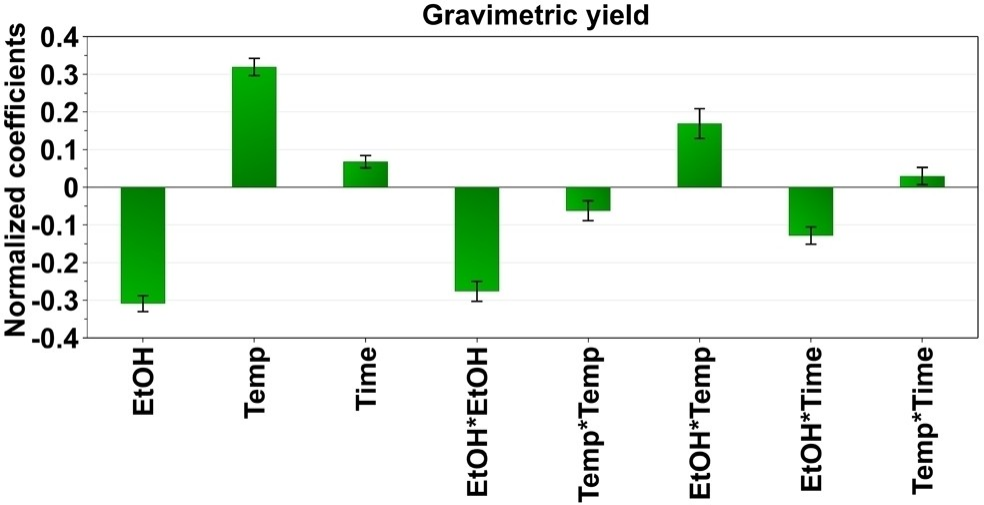
Coefficient plots from the Box–Behnken design showing the influence of different extraction parameters on the gravimetric yield (wt.%) obtained.

**Figure 3:**
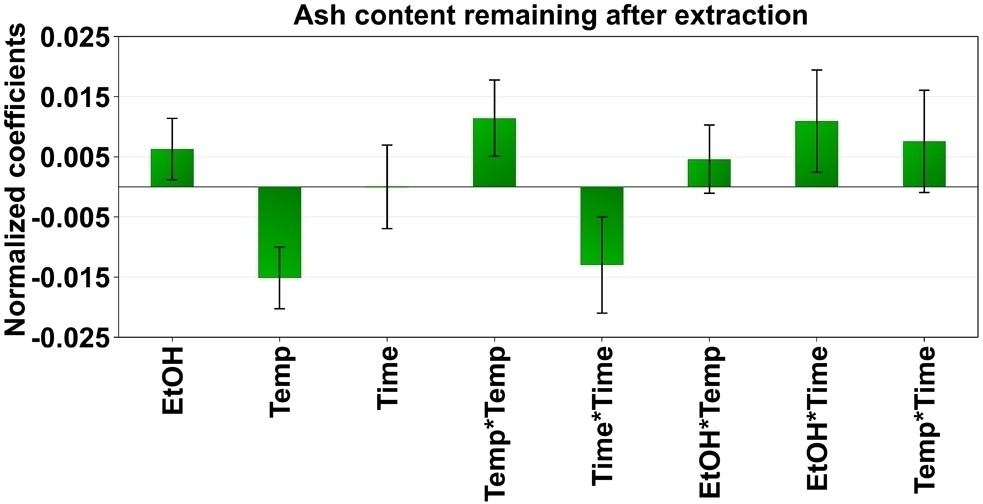
Coefficient plots from the Box–Behnken design showing the influence of different extraction parameters on the ash content remaining (wt.%) in the extracted coal.

Based on the extraction of raw coal by subcritical liquid extraction, an empirical relationship between experimental results obtained and input variables was established by fitting a second‐order polynomial equation with interaction terms and applying multiple linear regression. The fitted model showed a total explained variance of 97–99 % (R^2^=0.97–0.99) that indicated an excellent model fit. An estimation of the future prediction and precision is shown by the cross‐validated predictability of 53–93 % (Q^2^=0.53–0.93). The validity of the model and its capabilities to predict the best extraction conditions within the experimental design domain are highlighted by the linearity of the predicted vs. observed value plot **(**Figure S1 in Supporting Information). Coefficient plots for desulfurization, gravimetric yield and demineralization are shown in Figures 1, 2, and 3, respectively.

### Optimum extraction conditions for desulfurization of the coal

**Figure 4:**
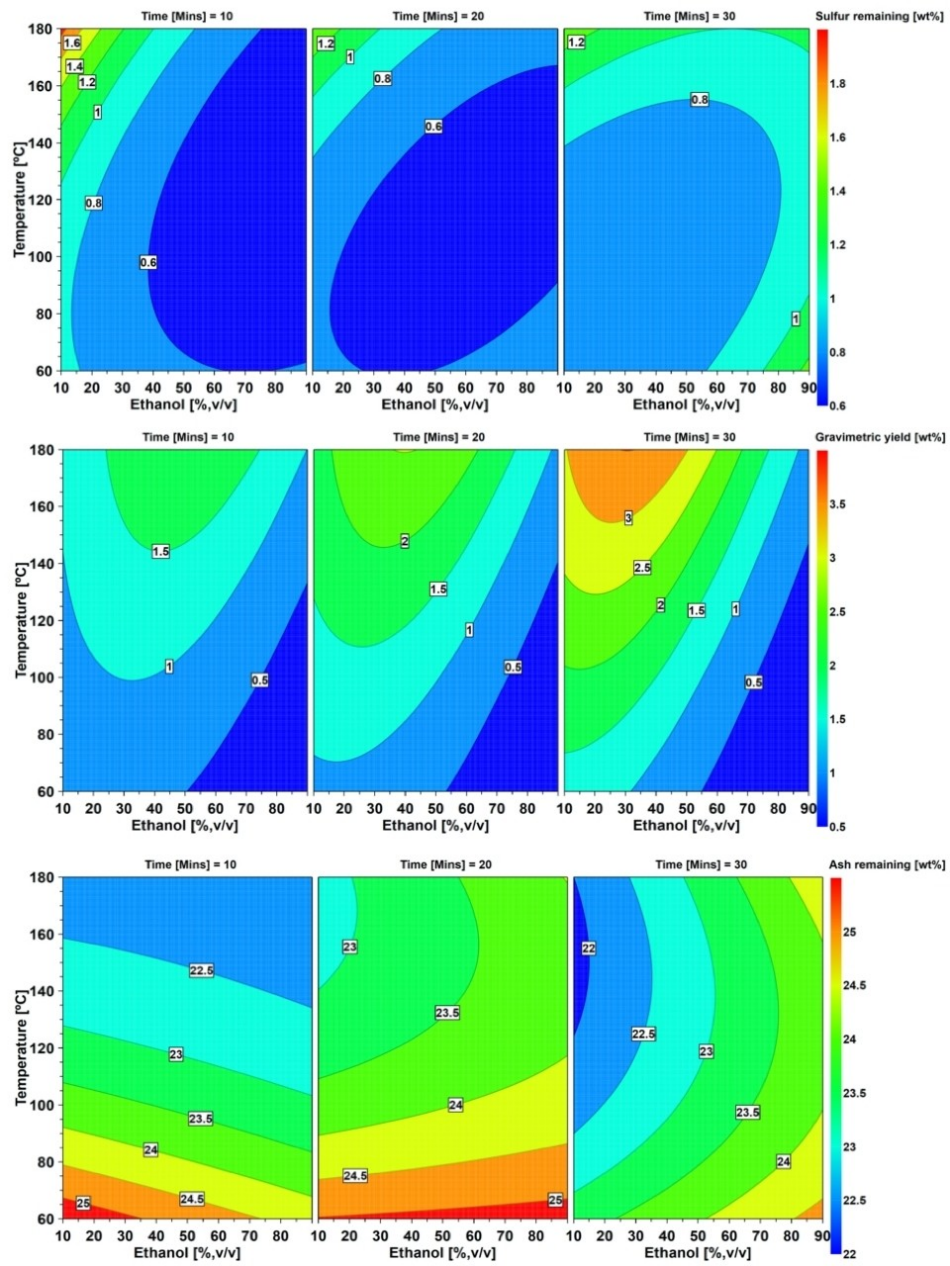
Contour plots showing the effects of temperature (°C), extraction time (mins,) and ethanol (%, v/v) on the gravimetric yield (%), and the ash content (wt.%) as well as sulfur content (wt.%) remaining in the Morupule coal after the extraction.

Inorganic forms of sulfur such as sulfate and sulfide dominate in coal.^[45]^ Organic sulfur is found in different forms such as thiolic (R‐SH), sulfoxidic (R_2_S‐O), sulfidic (R‐S‐R′), disulfidic (R‐S‐S‐R) and thiophenic (heterocyclic).^[46]^ A study conducted by Azzam and Sunggyu showed a reduction from 7.1 to 6.1 (wt.%) of total sulfur, extracted at 400 °C and 136 bars for 1 h with a supercritical ethanol/water (16/84 %, v/v) mixture. They quantified the sulfur by following the ASTM D‐2492 testing method.^[21]^ In our study, as already mentioned, we also studied the effect of different proportions of water/ethanol in the solvent. In addition, we explored relatively mild temperatures between 60 and 180 °C. The aim herein was to achieve minimum sulfur content remaining in the coal after extraction (close to 0 %). The coefficient plot is shown in Figure 1. Parameters with positive coefficients show that increasing their amounts results in less sulfur removal from coal. The negative coefficient of ethanol indicates that increasing ethanol content results in the decrease in the sulfur content in the extracted coal and the interaction of ethanol and temperature results in high extraction efficiency of sulfur from the coal. This observation can be visually seen in the contour plots (see Figure 4), demonstrating that ethanol negatively influences the sulfur remaining in the coal. Increasing the extraction temperature with increasing ethanol content results in an enhanced reduction in the sulfur content. Our study reports a total sulfur reduction from 1.9 to 0.4 (wt.%) at optimum conditions.

Based on the optimizer function that take into account the effect of the interaction on the reduction of sulfur in coal, the optimized extraction conditions for maximum desulfurization of the coal were 10 min of extraction time with 90 % ethanol in water at 129 °C and 105 bars. It is not possible to distinguish between the different types of sulfur being extracted because in our study the extracted coal is acid digested prior to analysis by inductively coupled plasma optical emission spectroscopy (ICP‐OES). The ASTM D‐2492 testing method should be used to determine the amounts of different kinds of sulfur.[^47^, ^48^] In our study, digestion of the whole coal sample was carried out with an ionic acid combination of HCl, HNO_3_ and HF for complete decomposition. Microwave heating ensured a minimal loss of volatile sulfur since it uses a closed system. Laban and Atkin applied the above mentioned method to bituminous and sub‐bituminous coals and their method was validated with good precision.^[49]^

### Optimum extraction conditions for gravimetric yield

The efficiency of polar and non‐polar solvents for extraction of organic compounds from coal is reported in the literature. In general, the extraction yield for organic compounds varies with the polarity of the solvent and the temperature of extraction. For example, Shui et al. showed the highest extraction yield of Chinese sub‐bituminous coal (Shenfu coal) was 56 wt.% obtained at 360 °C with 1‐methylnaphthalene. In contrast, Kim et al. (2008) studied the extraction of organic components from Roto South and Kideko sub‐bituminous coals and Sunhwa bituminous coal with N‐methyl‐2‐pyrrolidinone (NMP). Roto South coal extraction yield reached 70 wt.% at 350 °C and 85 wt.% at over 400 °C. The extraction yield of Kideko coal reached 67 wt.% at 430 °C whereas Sunhwa coal extraction yield reached 65 wt.% at 400 °C with 60 wt % 350 °C.^[16]^ Other solvents that have been studied are ethylenediamine (EDTA),^[50]^ methanol,^[51]^ and ethanol.^[52]^

In our study, we applied a mixture of water and ethanol in different ratios (1 : 5–5 : 1, v/v), at different temperatures (60–180 °C), and with varying times of extraction (10–30 mins). A Box–Behnken design was used to optimize the method. The effects of the set of independent variables on the gravimetric yield were investigated by observing the coefficient and the contour plots. The coefficient plot for gravimetric yield in Figure 2 shows that ethanol in water significantly reduced the total yield of extractable material in the coal. At the same time, the temperature significantly increased the extraction yield, as did the extraction time. Figure 4 shows that the contour plots highlight that the highest temperature investigated (180 °C), and the highest extraction time applied (30 min) resulted in the highest yield of extractable matter within the experimental domain. The ethanol content for maximum yield is less than 50 %, as seen in the contour plots at the highest temperature and extraction time. The results suggest that to obtain the highest gravimetric yield, some ethanol is needed – most likely to help reduce the viscosity of the solvent and in wetting of the coal matrix to improve diffusion.[^53^, ^54^] The optimal conditions for maximum extraction of solutes were determined by the DoE optimizer function, and turned out to be 30 vol % ethanol in water, a temperature of 180 °C, and an extraction time of 30 minutes

### Optimum extraction conditions for the reduction of mineral matter

The mineral matter content of the raw coal under study was determined to be 24.4 wt.%, reported as ash content (see Table 1). A high amount of mineral matter in coal is undesirable as described in the Introduction. In this study, the effect of ethanol content in water, extraction time and temperature were investigated as a function of mineral matter remaining in the coal after the extraction. The coefficient plot for the reduction of mineral matter measured as ash remaining in the extracted coal (see Figure 3) shows that the addition of ethanol to water significantly increases the removal of ash, although the effects are minor. In general, most parameters give no significant or just small impact on the amount of ash remaining in the coal within the studied experimental range. It has been reported that the temperature positively influences the demineralization of coal both by acid/base leaching and solvent extraction, which is attributed to thermally induced and/or solvent‐induced relaxation of the covalent bonds that hold coal molecules together.[^55^, ^56^, ^57^] It should be noted that the temperatures used in our study did not result in the thermal degradation of coal. Based on preliminary experiments carried out by thermogravimetric analysis (TGA), thermal decomposition starts at around 380 °C (TGA curves not shown but available on request).

The contour plots in Figure 4 show that higher temperatures and longer extraction times enhance the reduction in mineral matter. In contrast, the opposite is observed for ethanol content in the extraction solvent. Noticeably, the effects are minor. The optimized extraction parameters for maximum reduction of mineral matter were 30 min of extraction with 10 vol % of ethanol in water at 152 °C (105 bars).

### Predicted extraction conditions and recovery of sulfur by subcritical aqueous ethanol extraction

An additional set of experiments was performed with the predicted parameters to further validate the model. The total sulfur content that remains in the extracted coal was compared with the total sulfur content of the raw coal (before extraction) and the ash content before and after extraction (Table 2). The obtained results show that the predicted results are within the standard deviation of the experimental value. Ultimately, it means that the partial least square model can predict the extraction conditions quite accurately.

**Table 2:** Predicted vs. observed values of gravimetric yield (wt.%), ash remaining after extraction (wt.%), and total sulfur remaining after extraction (wt.%) of Morupule coal at optimum extraction conditions as defined by vol% of ethanol in water, temperature, and extraction time. n=3 at 95 % confidence interval.

Extraction method	Optimized method parameters			Response	Raw coal [weight %]	Predicted post‐extraction [weight %]	Observed post‐extraction [weight %]
	Ethanol [vol %]	Temp. [°C]	Time [mins]				
Desulfurization	90	129	10	Sulfur content	1.9±0.2	0.43±0.02	0.4±0.3
Gravimetric yield	31	180	30	Gravimetric mass	100	3.1±0.2	3.4±0.8
Demineralization	10	152	30	Ash content	24.4±0.4	21.9±0.3	23.0±0.3

## Conclusion

The present study shows how a Box–Behnken design may be efficiently applied to develop an extraction method to reduce the total sulfur content in raw coal. At optimum conditions (temperature=129 °C, ethanol=90 %, extraction time=10 minutes), a 79 % reduction was achieved. The optimized parameters for a maximum reduction of ash were found to be 10 % ethanol in pressurized hot water (105 bars) at 152 °C within 30 minutes, giving a 5 % reduction in ash content. We have thus, shown that a pressurized mixture of hot water and ethanol has potential as a green solvent. Even though in our study we have not performed the analytical eco‐scale for assessing the greenness of the desulfurization extraction method,^[58]^ we do use well‐established greener solvents^[59]^ under relatively mild conditions without thermal degradation of coal. With regards to the total sulfur extraction efficiency of the here developed method, the extraction temperature is lower (129 °C) and the extraction time is shorter (10 minutes) compared to a previously reported method for desulfurization of Midwestern U.S. bituminous coal designated as Indiana 5 that used an ethanol/water (35/65 vol %) mixture at temperatures of 400 °C and 60 minutes extraction time.^[21]^ We believe that the proposed extraction method could be effective in the extraction of other coals in Botswana and similar coals elsewhere. Further work needs to be carried out to characterize the types of sulfur (organic and inorganic) and determine the selectivity of the extraction method. In addition, the optimized conditions for the extraction of sulfur from coal were less effective in reducing ash content. Additionally, we anticipate the evaluation of the extraction method on extraction of nitrogen species in the coal. Furthermore, there is a need to evaluate the greenness of the proposed method and explore the up‐scaling of the method.

## Experimental Section

**Chemicals and samples**. Ethanol (99.7 %) was purchased from Solveco, Rosenberg, Sweden. Formic acid (95 %), metals used as standards and Whatman® Puradisc (0.22 mM) syringe filters were purchased from Sigma Chemical Co. (St. Louis, MO, USA). A Milli‐Q, Millipore system (Merck, Massachusetts, USA) was used to generate ultrapure water (18.2 MΩ cm) used for the preparation of standards and in extraction experiments. Coal samples were collected from Morupule Colliery (22°30′25.5 “S 27°01′35.6 ”E). The coal samples were crushed to pass through a 200‐mesh sieve and stored in the freezer (−4 °C) before analysis. Table 1 presents the conventional chemical analyses (proximate and ultimate analyses) of the raw coal samples, which were carried out at SGS laboratories (SGS (Pty) Ltd) in South Africa using the American Society for Testing and Materials (ASTM) standards. Determination of mineral oxides in the coal ash using ASTM D3174 method performed using a Bruker S8 Tiger XRF and Spectra Plus Version 3.0.2.19 (© 2012 Bruker AXS GmbH) was used for data processing.

**Experimental Design**. The influence of static extraction time, extraction temperature, and percentage of ethanol in water under subcritical conditions on the extraction of Morupule coal compounds were investigated using a response surface methodology (RSM) based on three levels. The experimental design was based on a Box–Behnken design (MODDE 10.1, Sartorius Stedim Biotech, Malmö, Sweden) with 4 central points and a total of 16 experiments. An ethanol volume fraction (X_1_) between 10 and 90 % (v/v) with an extraction temperature (X_2_) ranging from 60 to 180 °C, and an extraction time (X_3_) of 10 to 30 minutes, were the independent variables. The responses (dependent variables) were extraction yield (Y), ash content (A) remaining in the extracted coal, and the sulfur content (S) remaining in the coal after extraction, respectively. The unit of all responses was weight percent (%) in relation to the initial raw coal sample mass. The contour plots and model fits were calculated with multilinear regression. The adequacies of the models were evaluated by the R^2^ showing the model fit, and Q^2^ indicating an estimate of the prediction precision (predicted vs. observed) plots, and coefficient plots. Numerical and graphical analysis based on the criteria of the desirability function and response surface plots were used to determine the optimum processing conditions for the maximum extraction of sulfur from coal and the maximum reduction in the ash content of the coal.

**Subcritical liquid extraction and analysis methods**. An accelerated solvent extractor (ASE 350, Dionex, Sunnyvale, CA, USA), equipped with a solvent controller, was used to perform extractions. A 10 mL stainless steel vessel was loaded with 2.5 g of the raw coal sample for each extraction. Semi‐continuous extraction was done according to specified conditions as indicated in Table 3. The cell was loaded into the oven and filled with solvent until a pressure of 105 bar was reached then the pump automatically stopped pumping. The pressure is maintained by a closed static valve located below the cell. The cell was the heated to the desired temperature in 5 minutes. After each extraction, the solvent was purged from the cell with nitrogen gas. Between each extraction, the system was rinsed with the extraction solvent. The aqueous extracts were filtered through 0.22 mm nylon filter paper before evaporating the solvent under a gentle stream of nitrogen to determine the gravimetric yield. The gravimetric yield was calculated by weighing the extracted amount and subtracting it from the original amount of coal used for extraction.

**Table 3:** Experimental conditions of the Box–Behnken design and the corresponding gravimetric yield (Y), the ash content remaining in the extracted coal (A), and sulfur content remaining in the extracted coal (S).

Exp No	Run order	X_1_^[a]^ [%]	X_2_^[b]^ [°C]	X_3_^[c]^ [mins]	Y [wt.%]	A [wt.%]	S [wt.%]
1	6	10	60	20	0.9	25	0.6
2	12	90	60	20	0.1	25	0.7
3	13	10	180	20	1.8	23	0.7
4	15	90	180	20	0.7	24	0.8
5	8	10	120	10	0.9	23	0.5
6	5	90	120	10	0.4	24	0.5
7	4	10	120	30	2.2	22	0.5
8	14	90	120	30	0.3	24	0.4
9	2	50	60	10	0.5	25	0.7
10	16	50	180	10	1.9	33	0.9
11	3	50	60	30	0.6	24	0.5
12	10	50	180	30	3.0	23	0.9
13	9	50	120	20	1.4	24	0.9
14	7	50	120	20	1.3	24	1.0
15	1	50	120	20	1.3	24	0.7
16	11	50	120	20	1.4	24	0.7

[a] Ethanol content (v/v), [b] temperature (°C), [c] extraction time (min).

Before and after subcritical liquid extraction, coal samples were digested in a Marc 5 CEM microwave digester (CEM Corporation, North Carolina, USA). Firstly, 1 g of the extracted coal was ashed following the ASTM D3174^[41]^ at 500 °C for 1 h. The temperature was then increased to 750 °C and held for 1 h. Approximately 0.2 g of the ashed coal was digested in a mixture of 10 mL of distilled water, 6 mL of nitric acid (65 %), 2 mL of hydrochloric acid (37 %), and 2 mL of hydrofluoric acid (40 %) in a microwave digester, in a closed vessel. The digestion was carried out at temperatures ramping from room temperature (25 °C) to 180 °C for 20 min and held at 180 °C for 30 min. The samples were then diluted to 50 mL with 1.5 % boric acid and analyzed for total sulfur content on an inductively coupled plasma optical emission spectrometer (ICP‐OES), Optima 8300 from Perkin Elmer.

## Conflict of interest

The authors declare no conflict of interest.

1

## Supporting information

As a service to our authors and readers, this journal provides supporting information supplied by the authors. Such materials are peer reviewed and may be re‐organized for online delivery, but are not copy‐edited or typeset. Technical support issues arising from supporting information (other than missing files) should be addressed to the authors.

Supporting Information

## Data Availability

The data that support the findings of this study are available from the corresponding author upon reasonable request.
