# Supplementary material for: Desulfurization of Morupule Coal with Subcritical Aqueous Ethanol Extraction
Source: ChemistryOpen. 2022 Aug 10;11(11):e202200046. doi: 10.1002/open.202200046 (PMC9630044; doi:10.1002/open.202200046)
Supplement: Supplementary file 1 — Supporting Information [file OPEN-11-e202200046-s001.pdf]

ChemistryOpen

Supporting Information

Desulfurization of Morupule Coal with Subcritical Aqueous Ethanol Extraction

Fiona M. Nermark, Mmilili M. Mapolelo, James Darkwa, Ola F. Wendt, and Charlotta Turner*

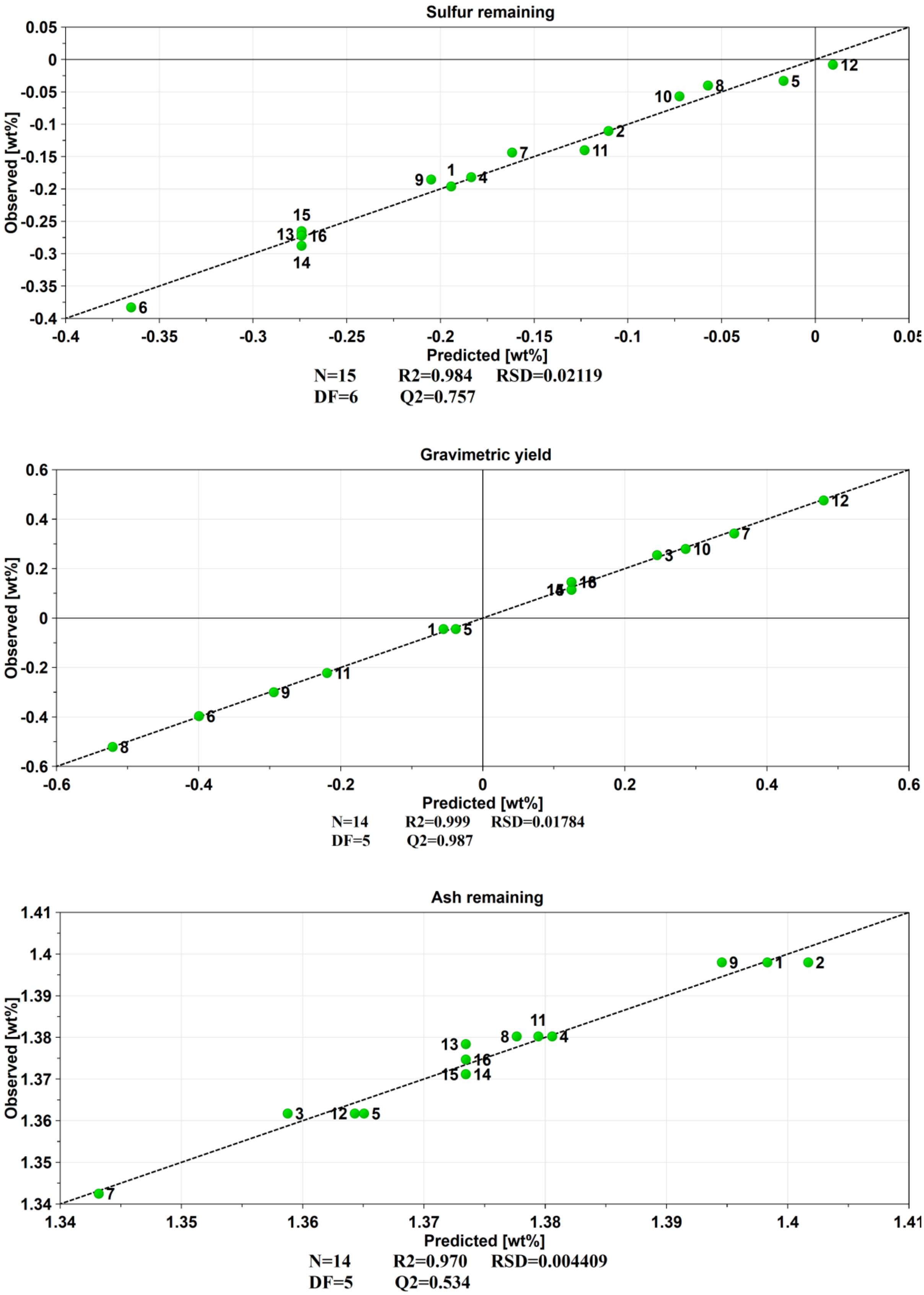

Figure S1. Plots showing the linear relationship between observed and predicted values from the models for gravimetric yield, ash content remaining in the coal and sulfur remaining in the coal after extraction.
